# Loss of Y Chromosome and Cardiovascular Events in Chronic Kidney Disease

**DOI:** 10.1161/CIRCULATIONAHA.124.069139

**Published:** 2024-09-03

**Authors:** Michael Weyrich, Sebastian Cremer, Martin Gerster, Tamim Sarakpi, Tina Rasper, Stephen Zewinger, Sammy R. Patyna, David M. Leistner, Gunnar H. Heine, Christoph Wanner, Winfried März, Danilo Fliser, Stefanie Dimmeler, Andreas M. Zeiher, Thimoteus Speer

**Affiliations:** 1Goethe University Frankfurt’s University Hospital, Department of Internal Medicine 4, Nephrology (T.S., S.R.P., M.G., T.S., M.W.); Else Kroener-Fresenius Center for Nephrological Research (T.S., S.R.P., M.G., T.S., M.W.); University Hospital, Department of Medicine, Cardiology (S.C., D.M.L.); Institute for Cardiovascular Regeneration (T.R., S.D., A.M.Z.), Germany.; German Center for Cardiovascular Research DZHK, Berlin, Germany (S.C., D.M.L., S.D., A.M.Z.).; Hôpital Robert Schumann, Hôpital Kirchberg, Luxembourg City, Luxembourg (S.Z.).; Saarland University, Department of Internal Medicine 4, Homburg/Saar, Germany (D.F., G.H.H., S.Z.).; University of Wuerzburg, University Hospital, Department of Clinical Research and Epidemiology, Comprehensive Heart Failure Center, Germany (C.W.).; University of Heidelberg, University Medical Center, Medical Faculty Mannheim, Vth Department of Medicine, Germany (W.M.).; Medical University of Graz, Clinical Institute of Medical and Chemical Laboratory Diagnostics, Austria (W.M.).; SYNLAB Holding Deutschland GmbH, SYNLAB Academy, Mannheim, Germany (W.M.).

**Keywords:** chronic kidney disease, heart failure, loss of Y-chromosome, LOY

## Abstract

**BACKGROUND:**

Chronic kidney disease represents one of the strongest risk factors for cardiovascular diseases, and particularly for heart failure. Despite improved pharmaceutical treatments, mortality remains high. Recently, experimental studies demonstrated that mosaic loss of Y chromosome (LOY) associates with cardiac fibrosis in male mice. Since diffuse cardiac fibrosis is the common denominator for progression of all forms of heart failure, we determined the association of LOY on mortality and cardiovascular disease outcomes in patients with chronic kidney disease.

**METHODS:**

LOY was quantified in men with stable chronic kidney disease (CARE for HOMe study, n=279) and dialysis patients (4D study, n=544). The association between LOY and mortality, combined cardiovascular and heart failure-specific end points, and echocardiographic measures was assessed.

**RESULTS:**

In CARE for HOMe, the frequency of LOY increased with age. LOY >17% was associated with increased mortality (heart rate, 2.58 [95% CI, 1.33–5.03]) and risk for cardiac decompensation or death (heart rate, 2.30 [95% CI, 1.23–4.27]). Patients with LOY >17% showed a significant decline of ejection fraction and an increase of E/E’ within 5 years. Consistently, in the 4D study, LOY >17% was significantly associated with increased mortality (heart rate, 2.76 [95% CI, 1.83–4.16]), higher risk of death due to heart failure and sudden cardiac death (heart rate, 4.11 [95% CI, 2.09–8.08]), but not atherosclerotic events. Patients with LOY >17% showed significantly higher plasma levels of soluble interleukin 1 receptor-like 1, a biomarker for myocardial fibrosis. Mechanistically, intermediate monocytes from patients with LOY >17% showed significantly higher C-C chemokine receptor type 2 expression and higher plasma levels of the C-C chemokine receptor type 2 chemokine (C-C motif) ligand 2, which may have contributed to increased heart failure events.

**CONCLUSIONS:**

LOY identifies male patients with chronic kidney disease at high risk for mortality and heart failure events.

**Clinical Perspective**

**What Is New?**

- Mosaic loss of Y chromosome increases with age and associates with mortality in men with chronic kidney disease.
- A higher frequency of loss of Y chromosome predicts heart failure end points in 2 cohorts comprising the entire spectrum from incipient chronic kidney disease to end-stage kidney disease.
- Loss of Y chromosome is associated with worsening left ventricular systolic and diastolic function, higher plasma concentrations of the myocardial fibrosis marker soluble interleukin 1 receptor-like 1, as well as activation of the chemokine (C-C motif) ligand 2 C-C chemokine receptor type 2 axis.

**What Are the Clinical Implications?**

- Loss of Y chromosome represents a novel risk factor for cardiovascular disease in patients with chronic kidney disease and may explain the higher risk for heart failure in men.
- Future research will show on whether these patients may benefit from specific therapeutic strategies as a personalized and sex-specific treatment approach in a growing population of the elderly.

Cardiovascular diseases (CVD) represent the leading cause of death in Western populations. Chronic kidney disease (CKD) has been identified as one of the strongest risk factors for CVD, which accounts for a substantial reduction of life-expectancy.^1^ For instance, a 30-year-old patient with an estimated glomerular filtration rate (eGFR) of 15 to 29 mL/min per 1.73 m^2^ lives approximately 30 years less than an age-matched subject with normal kidney function.^1^ Accordingly, the risk for death due to cardiovascular events is higher than reaching the end-stage of CKD requiring kidney replacement therapy.^2^ Mortality in patients with an eGFR <15 mL/min per1.73 m^2^ is 5.9-fold higher as compared to subjects with an eGFR ≥60 mL/min per 1.73 m^2^.^3^ The spectrum of CKD-associated CVD includes atherosclerotic diseases such as myocardial infarction (MI), stroke, and peripheral artery disease, but also heart failure, arrhythmias, and sudden cardiac death.^1^ From 1990 to 2019, the number of disability-adjusted life years attributed to CKD particularly in the elderly increased by 130%.^4^ Moreover, the pooled prevalence of CKD in Western civilizations ranges around 10%,^5^ thus rendering CKD a global public health problem.

Among CVD risk, specifically the incidence of heart failure has been reported to increase with age and is higher in men as compared to women.^6^ This is related to a longer cumulative life-time exposure to traditional cardiovascular risk factors such as arterial hypertension or diabetes, but also to the presence of nonmodifiable cardiovascular risk factors during the aging process including the presence of somatic mutations in hematopoietic stem cells.^7,8^

Recently, mosaic loss of Y chromosome (LOY) in circulating white blood cells has been reported to occur with increasing age and to be associated with age-dependent diseases such as cancer, Alzheimer disease, and CVD in men.^9–11^ In population-wide samples, LOY has been detected in 20% of men and, therefore, represents the most frequently acquired somatic mutation in blood cells.^12^ Mechanistically, it has been shown that the presence of LOY predisposes to aging-associated cardiac fibrosis culminating in the development of heart failure in male mice.^13^ We have recently demonstrated that LOY in monocytes correlates with higher mortality in patients with severe degenerative aortic valve stenosis undergoing transcatheter aortic valve replacement in whom diffuse cardiac fibrosis is the major independent determinant of impaired survival even after successful valve replacement.^14,15^

Diffuse cardiac fibrosis is the common denominator for progression of all forms of heart failure^16^ and a major driver of heart failure in patients with CKD.^17^ Cardiac magnetic resonance imaging studies in patients with CKD indicate that myocardial fibrosis is already present in patients with early CKD and increases when kidney function deteriorates to the maximum in patients with end-stage kidney disease.^18^ These findings are in line with autopsy studies of human hearts of patients with CKD.^19^ Thus, we hypothesized that LOY may specifically interfere with the pathology of CKD-associated CVD. Consequently, it was the aim of the present study to quantify LOY in 2 well-designed cohorts of patients with CKD covering the entire spectrum from incipient CKD to end-stage kidney disease and to evaluate LOY as a sex-specific mediator of impaired outcome due to CVD in these patients.

## METHODS

Data are available from the corresponding authors upon reasonable request.

### CARE for HOMe Study

The CARE for HOMe study recruited 622 clinically stable patients with confirmed CKD at the nephrological outpatient clinic at the Saarland University Hospital, Homburg/Saar, Germany, between 2008 and 2014.^20^ The study was approved by the local ethics committee, and all participants provided written informed consent. CARE for HOMe included patients with CKD stage G2-4, according to the KDIGO guidelines (Kidney Disease Improving Global Outcomes), who were aged >18 years.^21^ Patients with active malignant disease, apparent infections, immunosuppressive therapy, patients after kidney transplantation, or acute kidney injury were excluded. Whole blood for DNA extraction was collected at study inclusion. eGFR was calculated using the CKD-EPI equation for creatinine and cystatin C.^21^ Echocardiographic measurements were performed at baseline as well as after 5 years by a single examiner unaware of biochemical results. Of the 622 participants, 335 were men. In 56 male participants, no DNA samples were available or available amount of DNA was too low for quantification of LOY. Accordingly, 279 subjects were included in the present analyses. Patients were followed for a median of 5.3 (4.0) years. Death refers to death from all causes. The combined cardiovascular end point comprises myocardial infarction, surgical or interventional coronary/cerebrovascular/peripheral arterial revascularization, stroke, amputation above the ankle, or death of any cause. The combined heart failure end point comprises hospital admission for decompensated heart failure (ie, symptoms of decompensated heart failure [dyspnea] together with clinical or radiological signs of heart failure) requiring intravenous diuretic treatment or death from any cause. The combined kidney end point comprises 50% reduction of eGFR or end-stage kidney disease (ie, requirement for renal replacement therapy).

No patient was lost to follow-up, and patients were censored at the time of the last annual follow-up visit in case of not experiencing an end point. The end points were adjudicated independently by 2 nephrologists blinded for the results. In case of disagreement, final decision was made by a third investigator.

### 4D Study

The 4D study is a multicenter, randomized, double-blind, prospective study including 1255 patients with type 2 diabetes receiving hemodialysis. Study details were previously described in detail.^22^ Patients were randomized to either atorvastatin (20 mg) or placebo. Written informed consent was obtained from all participants, and the study was approved by the institutional review committee. Of the 1255 participants, 677 were men. In 133 male participants, no DNA samples were available or available amount of DNA was too low for quantification of LOY. Accordingly, 544 subjects were included in the present analyses. Median follow-up time was 2.7 (2.4) years. Death refers to as death from all causes. The primary end point was the composite of death from cardiac causes, fatal stroke, nonfatal MI, or nonfatal stroke, whichever occurred first. One event per subject was included in the analysis. For the present analysis, we considered 2 additional ad hoc end points: (1) heart failure event (ie, the composite of death due to heart failure and sudden cardiac death) and (2) coronary event end point (ie, the composite of fatal MI, death during/after percutaneous transluminal coronary angioplasty, aortocoronary venous bypass, or other intervention due to coronary artery disease [CAD], death from other coronary causes, nonfatal MI, silent MI, nonfatal percutaneous transluminal coronary angioplasty, aortocoronary venous bypass, or other intervention due to CAD). End points were adjudicated by 3 members of the end point committee according to the study protocol. Members of the end point committee were blinded to the treatment.

### LOY Quantification

LOY was quantified as previously described^14^ using a validated polymerase chain reaction (PCR) technique in whole blood samples. Briefly, relative number of X and Y chromosomes in DNA samples was quantified using a TaqMan-based method targeting a 6 bp sequence difference present between the *AMELX* and *AMELY* genes using the same primer pair. Therefore, this method is relatively unbiased regarding the primer properties. PROBE PCR master mix (QIAcuity Probe PCR Kit, Qiagen, Germany) containing FastDigest HindIII enzyme (ThermoFisher) and TaqMan Primers (ThermoFisher) was mixed with 115 ng of DNA. After digestion (10 minutes, room temperature), a 26k 24-well Nanoplate was filled with the reaction mix and loaded into a QIAcuity ONE instrument. PCR was performed according to the manufacturer’s instructions: 95 °C for 2 minutes, 2-step cycling (40 cycles) for 95 °C for 15 seconds and 60 °C for 30 seconds. A 6 nt deletion occurs in the X-specific *amelogenin* gene (B37/hg19 genome locations: chrX:11315039 and chrY:6 737 949–6 737 954). The VIC dye probe detects X-chromosome sequences, and the FAM dye probe includes the 6 nt and detects Y-chromosome sequences (Sequence: GTGTTGATTCTTTATCCCAGATG[-/AAGTGG]TTTCTCAAGTGGTCCTGATTTT [VIC/FAM]).

End point fluorescence intensity of the partitions was separately measured for FAM (*AMELY*) and VIC (*AMELX*) to determine the presence/absence of the respective targets. The absolute concentration of the targets was calculated using the QIAcuity One Software suite (Qiagen, Germany) based on the number of positive and negative partitions to calculate the ratio of *AMELY*/*AMELX*. Extent of LOY was defined as percent of cells with LOY derived from the ratio *AMELY*/*AMELX*.

### Soluble Interleukin 1 Receptor-Like 1 and Chemokine (C-C Motif) Ligand 2 Enzyme-Linked Immunosorbent Assay

In CARE for HOMe, plasma levels of soluble interleukin 1 receptor-like 1 (sST2) and chemokine (C-C motif) ligand 2 (CCL2) were measured using commercially available enzyme-linked immunosorbent assays (R&D systems, #DST200 and #DCP00) according to the manufacturer’s protocol. In 4D, sST2 was measured using the Presage ST2 sandwich immunoassay (Critical Diagnostics).

### Flow Cytometry

Monocyte subpopulations in CARE for HOMe were quantified by flow cytometry as described previously.^23^ Briefly, 100 µL of whole blood was stained using anti-CD16 (Invitrogen) and anti-CD14 (BD Biosciences) antibodies and analyzed using a BD FACS Canto II flow cytometer. Monocyte populations were defined as classical (CD14^++^CD16^-^), intermediate (CD14^++^CD16^+^), and nonclassical (CD14^+^CD16^++^) monocytes. Mean fluorescence intensity of C-C chemokine receptor type 2 (CCR2) expression within every monocyte population was quantified using an anti-CCR2 (clone: Y15-488.rMAb, BD Bioscience) antibody. Analyses were performed using BD FACSDiva software.

### Statistical Methods

Continuous variables are presented as mean±SD when normally distributed or as median (interquartile range) for variables with skewed distribution. Categorical variables are presented as numbers and percentages. Homogeneity of variances was tested by using the Levene test. Statistical differences between continuous variables were tested using *t* test for normally distributed variables or Mann-Whitney test for not-normally distributed variables, as well as chi-square test for categorical variables. All observations were included in the present analyses.

Patients in CARE for HOMe and 4D were dichotomized at LOY of 17%, a cutoff for LOY, which was recently established using the Youden-index based on the area-under-the-curve from receiver-operating-curve analyses in patients undergoing transcatheter aortic valve replacement for severe degenerative aortic valve stenosis.^14^ Using the same approach, an optimal cutoff of 18% LOY was determined in CARE for HOMe. Since this cutoff was quite comparable to the 17% cutoff of LOY as established in our prior study,^14^ we decided to use the 17% cutoff in the present analyses to ensure comparability of the findings.

To prove that the association between LOY and different end points does not depend on a specific cutoff, additional models including LOY as continuous variable were built. The linearity assumption for quantitative predictors was assessed by visual inspection of the respective plots of the residuals versus the fitted values (not shown). The association between age and LOY in CARE for HOMe was visualized by using restricted cubic splines of age with 3 knots placed at 10th, 50th, and 90th percentile of age, respectively. The same method was applied to visualize the association between LOY and mortality in CARE for HOMe and 4D.

Cox proportional hazard models were built to assess the association between LOY and the different end points. Analyses in CARE for HOMe were adjusted for age, systolic blood pressure, BMI, diabetes, smoking, hsCRP, NT-proBNP, troponin T, CAD, eGFR, albuminuria, and urinary DKK3. Analyses in 4D were adjusted for age, systolic blood pressure, BMI, smoking, hsCRP, NT-proBNP, troponin T, CAD, congestive heart failure, and randomization group. hsCRP, NT-proBNP, and albuminuria were log-transformed. Survival plots showing the cumulative survival function are shown. The proportional hazard assumption for each variable in the respective Cox regression models was tested in STATA using the command “estat phtest, detail.” Linearity assumption for quantitative predictors was assessed by plotting the Martingale residuals against the quantitative predictors using the “predict mgale” command within the STATA “postestimation” command. To test the interaction between randomization group and LOY in 4D, a first order interaction term was included in the respective models. For the 2 additional ad hoc end points in 4D, we performed Bonferroni correction to the type I error rate. The predictive value of LOY in addition to clinical models (CARE for HOMe: age, systolic blood pressure, BMI, diabetes, smoking, hsCRP, NT-proBNP, troponin T, coronary artery disease, eGFR, albuminuria, and urinary *DKK3*; 4D: age, systolic blood pressure, BMI, smoking, hsCRP, NT-proBNP, troponin T, coronary artery disease, congestive heart failure, randomization group) was determined by integrated discrimination improvement and net reclassification improvement.

Generalized linear models (linear response, normal distribution, identity link function) were built to assess the association between LOY and changes of echocardiographic parameters as well as sST2 plasma levels and respective multivariable adjusted least square means were reported. Analyses in CARE for HOMe and 4D were adjusted for the parameters described above.

A 2-sided *P*<0.05 was considered statistically significant. Analyses were performed using SPSS version 21.0 and STATA IC 15 with the packages nriidi, postrcspline, estat phtest, and estat gof.

## RESULTS

### Associations of LOY With CVD Outcomes in CARE for HOMe

**Figure 1.**
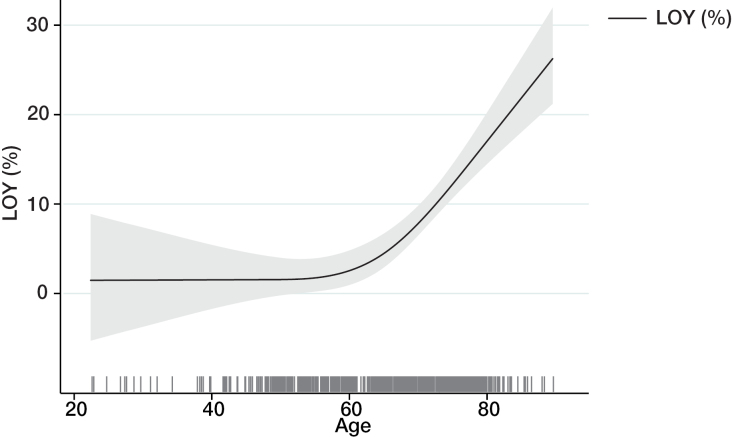
**Loss of Y chromosome (LOY) in the CARE for HOMe study.** Association between age and the frequency of LOY in men of the CARE for HOMe study. Line indicates LOY (%), with the 95% CI (gray area). Spikes show the individual distribution of age.

Loss of Y chromosome (LOY) was quantified in participants of the CARE for HOMe study. Figure 1A shows the association between LOY and age. There was a steep increase in the extent of LOY starting at the age of 60 years.

**Figure 2.**
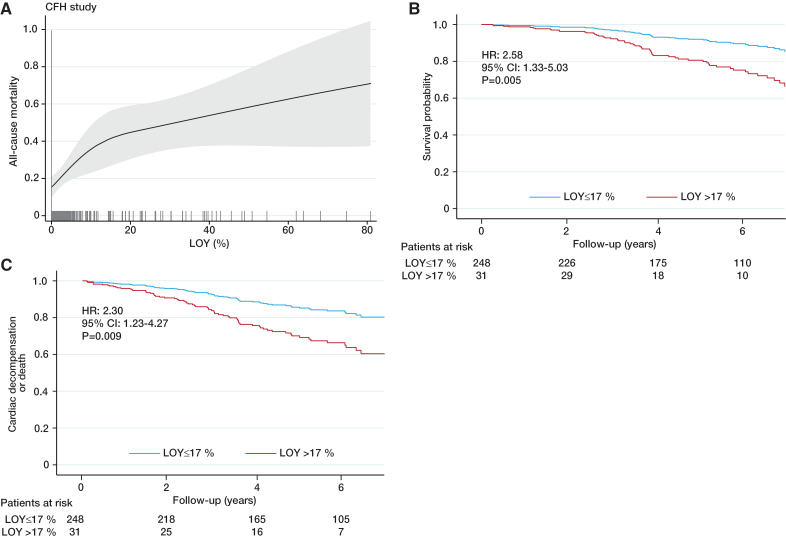
**Association between loss of Y chromosome (LOY), mortality, and cardiac decompensation in the CARE for HOMe study. A**, Restricted cubic spline plot on the association between LOY as continuous variable and all-cause mortality in 277 participants of the CARE for HOMe study. Plot is adjusted for age, systolic blood pressure, body mass index (BMI), diabetes, smoking, high sensitivity C-reactive protein (hsCRP), N-terminal pro-B-type natriuretic peptide (NT-proBNP), troponin T, coronary artery disease, estimated glomerular filtration rate (eGFR), albuminuria, and urinary *DKK3* (Dickkopf-3). **B**, Association between LOY and all-cause mortality as well as (**C**) the combination of cardiac decompensation or death in the CARE for HOMe study as determined by Cox regression analyses adjusted for age, systolic blood pressure, BMI, diabetes, smoking, hsCRP, NT-proBNP, troponin T, coronary artery disease, eGFR, albuminuria, and urinary *DKK3*. CFH indicates CARE for HOMe study; and HR, hazard ratio.

As shown in Figure 2A, the extent of LOY as a continuous variable was significantly associated with all-cause mortality after adjusting for a variety of potentially confounding variables (Table S1). Patients were dichotomized according to a recently described cut-off of >17% of LOY.^14^ Baseline characteristics of the CARE for HOMe study cohort are shown in Table 1. Participants with LOY >17% were significantly older, had a higher prevalence of CAD and CVD, a lower eGFR, and higher troponin T plasma levels. As illustrated in Figure 2B, LOY >17% was associated with a significantly higher all-cause mortality in participants of the CARE for HOMe study (heart rate [HR], 2.58 [95% CI, 1.33–5.03] *P*=0.005) after adjustment for important confounders including age and all differentially distributed parameters at baseline including NT-proBNP and troponin T (Table S2). As compared to a clinical model, addition of LOY significantly improved patient classification (Table S2).

**Table 1.**
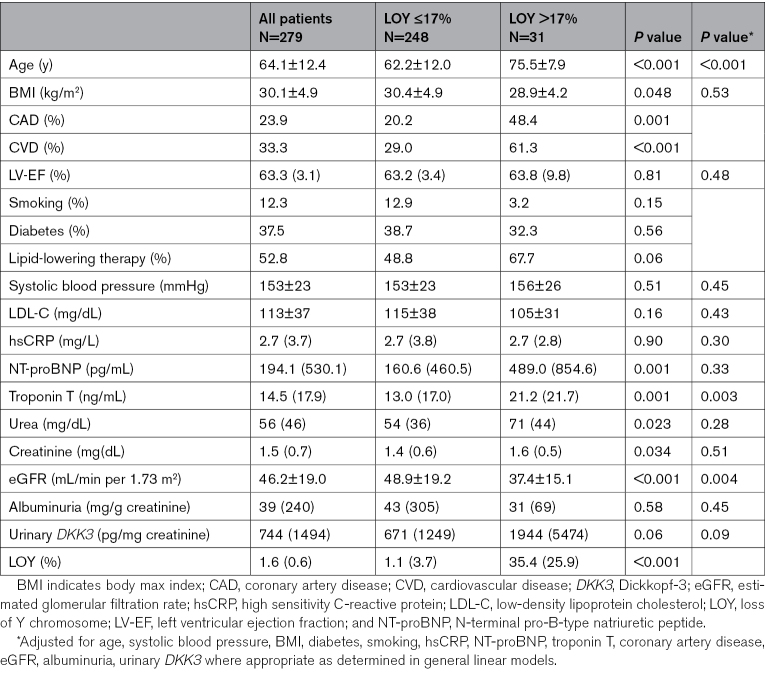
Baseline Characteristics According to Categories of LOY in Participants of the CARE for HOMe Study

We next determined the association between LOY and the combined cardiovascular end point. Compared to participants with LOY ≤17%, LOY >17% was associated with a significantly higher risk for the combined cardiovascular end point (MI, surgical or interventional coronary/cerebrovascular/peripheral arterial revascularization, stroke, amputation above the ankle, or death of any cause) in participants of CARE for HOMe (HR, 1.82 [95% CI, 1.04–3.20] *P*=0.037, Table S3) in multivariable adjusted models. These findings indicate that the higher mortality in participants with LOY >17% may be due to cardiovascular events. Importantly, as illustrated in Figure 2C, the increase in cardiovascular events in patients with LOY >17% was in large part driven by a significantly higher risk for decompensation of heart failure or death (HR, 2.30 [95% CI, 1.23–4.27] *P*=0.009, Tables S4 and S5) in the multivariable adjusted model.

**Figure 3.**
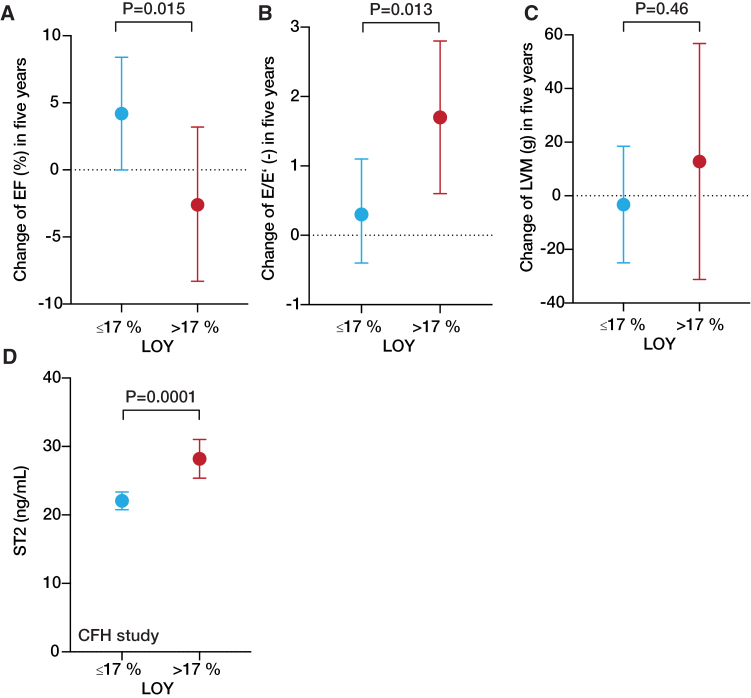
**Association between loss of Y chromosome (LOY) with echocardiographic measures and plasma levels of sST2 in the CARE for HOMe study.** Change of (**A**) ejection fraction (EF), (**B**) E/E’, and (**C**) left ventricular mass (LVM) within 5 years. **D**, sST2 plasma levels in participants of the CARE for HOMe study. Shown are the multivariable adjusted for age, systolic blood pressure, body mass index, diabetes, smoking, high sensitivity C-reactive protein, N-terminal pro-B-type natriuretic peptide, troponin T, coronary artery disease, estimated glomerular filtration rate, albuminuria, and urinary *DKK3* (Dickkopf-3) least square means (with 95% CI) as determined by general linear models.

To further corroborate these findings, we assessed the association between LOY and changes of echocardiographic markers in participants of CARE for HOMe (Figure 3A through 3C). Most importantly, LOY >17% was associated with a significant decline of multivariable adjusted least square means of ejection fraction and an increase of E/E’, but not left ventricular mass. In addition, it should be noted that LOY remained an independent predictor of mortality and cardiac decompensation/death in CARE for HOMe even after accounting for the presence of indicators of more severe heart failure as assessed by left ventricular ejection fraction as well as logical volume management (Table S6). Furthermore, patients with LOY >17% showed significantly higher plasma concentrations of sST2 (Figure 3D), a biomarker for myocardial fibrosis.^24^

In contrast, LOY did not associate with the combined kidney end point (ie, 50% reduction of eGFR or end-stage kidney disease, Table S7) or end-stage kidney disease end point (Table S8).

Thus, taken together, these data indicate that LOY associates with significant deterioration of both systolic and diastolic left ventricular function during 5 years of follow-up, which may account for the observed increase in heart-failure driven events leading to increased cardiovascular mortality.

### Associations of LOY With CVD Outcomes in the 4D Trial

After establishing a significant association between LOY and clinical outcomes in the CARE for HOMe study, we assessed the association between LOY and distinct outcomes in men enrolled in the randomized, controlled 4D study comprising dialysis patients, who were randomized to either atorvastatin or placebo.^22^ Baseline characteristics of men in the 4D trial are summarized in Table S9. Patients with LOY >17% were significantly older and the prevalence of smokers as well as congestive heart failure was higher as compared to subjects with LOY ≤17%.

**Figure 4.**
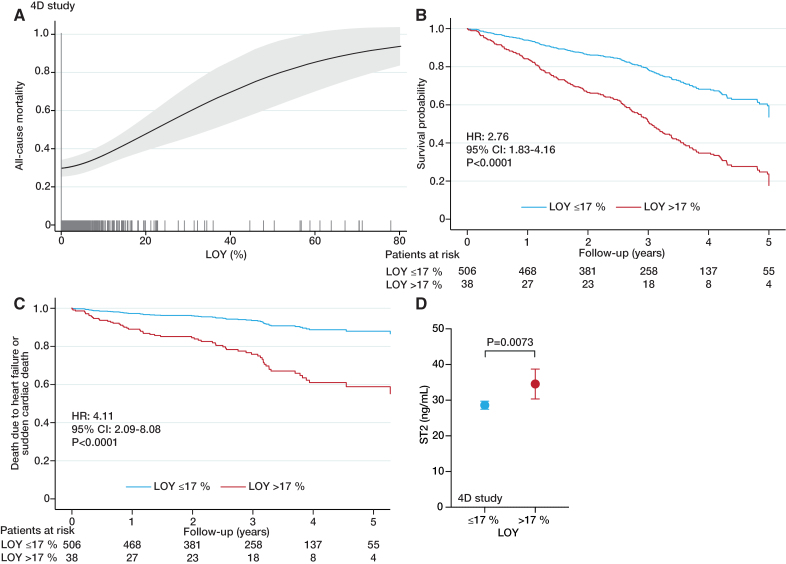
**Association between loss of Y chromosome (LOY), mortality, and heart failure events in the 4D study. A**, Restricted cubic spline plot on the association between LOY as continuous variable and all-cause mortality in the 4D study. Plot is adjusted for age, systolic blood pressure, body mass index (BMI), smoking, high sensitivity C-reactive protein (hsCRP), N-terminal pro-B-type natriuretic peptide (NT-proBNP), troponin T, coronary artery disease, congestive heart failure and randomization. **B**, Association between LOY and all-cause mortality in 544 participants of the 4D study as determined by Cox regression analysis adjusted for the same variables as in (A). **C**, Association between LOY and the combination of death due to heart failure and sudden cardiac death in the 4D study. Plot is adjusted for the same variables as in **A**. **D**, Multivariable adjusted (for age, systolic blood pressure, BMI, smoking, hsCRP, NT-proBNP, troponin T, coronary artery disease, congestive heart failure, and randomization) least square means (with 95% CI) of sST2 plasma levels in participants of the 4D study as determined by general linear models. Heart rate indicates hazard ratio.

In agreement with the results from CARE for HOMe study, LOY >17% was associated with a significantly higher all-cause mortality also in participants of the 4D study (HR, 2.76 [95% CI, 1.83–4.16] *P*<0.0001) Table S10, Figure 4A and 4B) after adjustment for important confounders. Likewise, the association between all-cause mortality and extent of LOY was independent of the selection of a specific cutoff of LOY as shown in Figure 4A, illustrating the association between extent of LOY as continuous variable and all-cause mortality (Table S11).

Consistently, patients in 4D with LOY >17% demonstrated a significantly higher risk for the combined cardiovascular end point, which slightly differed from the CARE HOMe study by including death from cardiac causes, fatal stroke, nonfatal MI, or nonfatal stroke (HR, 2.77 [95% CI, 1.76–4.36] *P*<0.0001, Table S12) in multivariable adjusted models.

Finally, death due to heart failure events and sudden cardiac death significantly contributed to the increased mortality observed in men in 4D with LOY >17% (HR, 4.11 [95% CI, 2.09–8.08] *P*<0.0001), multivariable adjusted model, Table S13), as illustrated in Figure 4C. In contrast, LOY was not associated with coronary events in the 4D study (Table S14). Accordingly, there was no interaction between LOY and randomization to either atorvastatin or placebo (*P*=0.89 for interaction, Table S15). In line with CARE for HOMe, LOY >17% was consistently associated with higher plasma levels of sST2 in 4D as well (Figure 4D). These findings further support the hypothesis that, in patients with CKD, LOY may contribute to the progression of heart failure and heart failure-driven events, but not of atherosclerotic CVD.

Table 2 illustrates the concordance of CARE HOMe study and the 4D trial with respect to the different end points analyzed. Furthermore, we found that the incidence of mortality as well as the heart failure end point was comparable between women and men with LOY ≤17%, but substantially higher in men with LOY >17% (Figure S1).

**Table 2.**
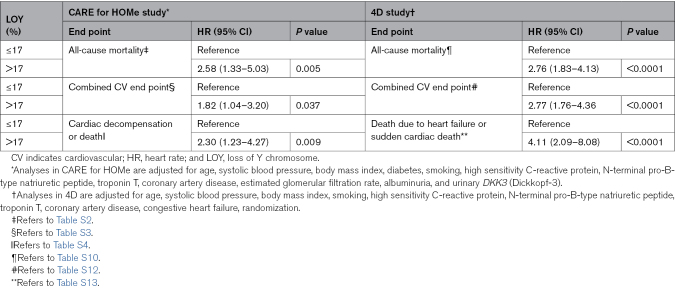
Summary on the Association Between LOY and Distinct End Points in the CARE for HOMe and the 4D Study

### Association Between LOY and Monocyte CCR2 Expression

**Figure 5.**
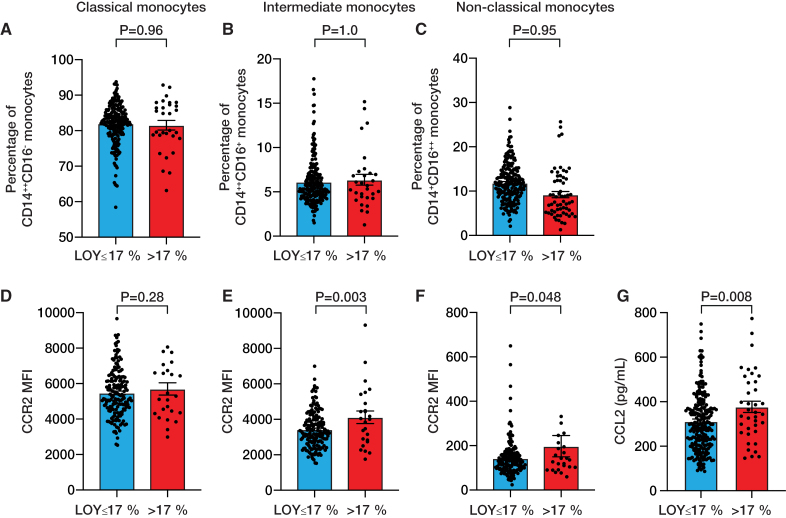
**Association between loss of Y chromosome (LOY) and monocyte CCR2 expression in the CARE for HOMe study.** Percentage of (**A**) classical (CD14^++^CD16^-^), (**B**) intermediate (CD14^++^CD16^+^), and (**C**) nonclassical (CD14^+^CD16^++^) monocytes according to LOY. Expression of CCR2 in (**D**) classical, (**E**) intermediate, and (**F**) nonclassical monocytes. **G**, Plasma levels of the CCR2 ligand CCL2 in participants of the CARE for HOMe study. Bars represent mean ± SEM.

Finally, to gain some first mechanistical insights on how LOY may contribute to CVD, we determined the frequency of monocyte subpopulations by flow cytometry in CARE for HOMe. We did not observe any difference in the frequency of classical, intermediate, or nonclassical monocytes according to LOY (Figure 5A through 5C). However, within the population of intermediate monocytes, we detected a significantly higher expression of the CCR2 chemokine receptor in intermediate monocytes from patients with LOY >17% as compared to those with LOY ≤17% (4179±270 vs. 3383±150, *P*=0.003, Figure 5D through 5F). In contrast, CCR2 expression did not differ in classical monocytes (*P*=0.276). Accordingly, plasma concentrations of the CCR2 ligand CCL2 were significantly higher in patients with LOY >17% (Figure 5G).

## DISCUSSION

The results of the present study for the first time demonstrate that LOY associates with increased cardiovascular mortality in patients with CKD. LOY was associated with cardiac decompensation and mortality due to heart failure events in 2 well-characterized cohorts comprising the entire spectrum of CKD patients from mildly impaired kidney function to patients with end-stage kidney disease. Therefore, LOY may represent a novel risk factor for cardiovascular events and may aid in the identification of CKD patients, who particularly benefit from preventive therapeutic strategies.

Previously, Sano et al,^13^ using a 40% LOY cut-off level in a large dataset derived from the UK biobank, reported a significant association of LOY with death due to hypertensive heart disease, aortic aneurysm and dissection, as well as to a lesser extent heart failure. However, the presence of CKD as a potential disease modifier and its prognostic interference with LOY was not addressed in the study by Sano et al.^13^ In the present analyses, we found that LOY was associated with heart failure events, but not with atherosclerotic cardiovascular events in patients with CKD. Accordingly, treatment with atorvastatin, which mainly exerts antiatherogenic effects,^1^ did not modify the association between LOY and mortality in the 4D study. This result is in line with recent experimental and clinical findings. LOY in murine bone marrow cells led to spontaneous diffuse cardiac fibrosis and development of heart failure in mice at the age of 15 months, enhanced cardiac fibrosis after transverse aortic constriction and reduced survival of the animals.^13^ Clinically, we have previously documented a shift towards a profibrotic gene signature in monocytes lacking the expression of Y chromosome-encoded genes in patients with severe degenerative aortic valve stenosis undergoing transcatheter aortic valve replacement.^14^ Importantly, in the present study, LOY was associated with significant deterioration of left ventricular systolic as well as diastolic function during 5 years of follow-up supporting a prominent role for progression of heart failure to contribute to the observed increased mortality. Fibrosis is not only the common denominator of progression of all forms of chronic heart failure,^16^ but is also very well established to contribute to the pathogenesis of dysrhythmias.^25^ In fact, fibrosis was found to predict ventricular arrhythmias and/or sudden death in all forms of chronic heart failure.^26^ Elevated plasma levels of the profibrotic biomarker sST2 were shown to have important prognostic value in large randomized trials in patients with heart failure.^27^ Thus, the composite end points of progression of heart failure and death used in both studies of the present analysis as well as the significantly elevated plasma levels of sST2 may indeed support a role for LOY to contribute to increased fibrosis resulting in worse clinical outcome in patients with CKD.

Mechanistically, we found that intermediate monocytes from patients with LOY >17% showed significantly higher CCR2 expression as compared to monocytes from patients with LOY ≤17% and higher plasma concentrations of the CCR2 ligand CCL2. Notably, a higher frequency of intermediate, but not classical or nonclassical monocytes was previously shown to associate with worse cardiovascular outcome in patients with CKD.^23^ Moreover, experimental studies revealed a prominent role for CCL2 to stimulate cardiac fibrosis via recruitment of pro-inflammatory and fibrogenic macrophages expressing CCR2.^28^ CCR2-expressing monocytes and macrophages are crucially involved in adverse cardiac remodeling.^29^ CCR2^+^ monocytes differentiate into CCR2^+^ macrophages that infiltrate the heart after MI or pressure overload and induce pathological left ventricular remodeling and cardiac fibrosis.^30,31^ These findings suggest that LOY may not only associate with augmented monocyte CCR2 expression, but also increased CCL2-mediated homing signals, thereby inducing cardiac fibrosis to promote the incidence and progression of heart failure.

Nevertheless, the cellular consequences of LOY in circulating blood cells remain to be fully understood. LOY may disease-specifically affect T cells including NK cells as shown for patients with Alzheimer’s disease as well as CD4+ T lymphocytes in cancer patients.^32^ Very recently, an enrichment of LOY in regulatory T cells (Tregs) associated with alteration of immunosuppressive functions was described.^33^ Treg dysregulation has been linked to processes such as long-term inflammation, tissue damage and increased fibrosis.^34^ Specifically, dysfunctional and pro-inflammatory Tregs are essential for adverse cardiac remodeling and diffuse cardiac fibrosis.^35^ Thus, it will be important to address additional cellular mechanisms involved in mediating the adverse effects of LOY in circulating blood cells on heart-failure related outcome in patients with CKD.

While assessing the extent of LOY may provide for an important novel marker of risk stratification for cardiovascular events related to heart failure, the therapeutic consequences of the identification of CKD patients with LOY are yet unclear. Currently, treatment of patients with CKD at risk for heart failure includes angiotensin-converting enzyme inhibitors or angiotensin receptor-neprilysin inhibitors, mineralocorticoid receptor antagonists, and, more recently, sodium-glucose transporter-2 inhibitors. The latter have been shown to reduce heart failure events and cardiovascular mortality in patients with heart failure and with CKD.^36,37^ Similarly, novel nonsteroidal mineralocorticoid receptor antagonists, which are known to reduce cardiac fibrosis development, were recently shown to specifically reduce heart failure–related outcomes in patients with CKD and diabetes but do not reduce cardiovascular mortality.^38^ Thus, future studies have to determine as to whether patients with LOY will particularly benefit from distinct medications in CKD.

Our study is not without limitations. First, the CARE for HOMe and the 4D study mainly included participants of White ancestry. Therefore, our results cannot be generalized to other ethnicities. Second, our analyses are based on an optimized cut-off of 17% for LOY, which we applied in both studies. Nevertheless, additional analyses using LOY as a continuous variable confirmed the association between LOY, mortality, and cardiovascular end points indicating that the present results are not restricted to the selection of a specific cut-off. Third, while CARE for HOMe included patients across the entire range of CKD, the 4D study included patients with type 2 diabetes receiving dialysis. We purposely selected the 4D study as additional cohort for 2 reasons: to extend our findings to end-stage CKD patients, who do experience the worst prognosis with CKD, and, more importantly, to enrich our analyses for a potential role of diffuse cardiac fibrosis given that myocardial fibrosis is a hallmark of the pathogenesis of heart failure and arrhythmogenesis in patients with type 2 diabetes.^39^ Fourth, the primary end points in CARE for HOMe and 4D are slightly different. However, we do believe that building an additional end point in 4D comprising the composite of death due to heart failure or sudden cardiac death compares favorably with the composite end-point decompensation for heart failure or death in CARE for HOMe. In CARE for HOMe, the pre-defined combined end points include the component all-cause mortality, which may introduce a certain bias. Fifth, both patient cohorts analyzed were recruited before the introduction of SGLT2 (sodium-glucose transport protein 2) inhibitors into clinical practice. Thus, we cannot comment on a potential disease-modifying effect of this class of drugs and its potential interaction with LOY. Blood samples for monocyte evaluation as well as echocardiographic data were only available in CARE for HOMe, but not in 4D. Thus, our mechanistic insights into disease progression potentially underlying the observed increase in heart failure related events could not be prospectively replicated in 4D. For measuring the extent of LOY, we used a previously validated PCR technique, which was shown to provide a close to perfect fit to a linear regression line (r^2^=0.998, n=26) compared to whole genome sequencing as the standard method to assess LOY.^40^ Thus, it is unlikely that spontaneous deletion of the 6 bp intergenic region amplified by the PCR may have confounded our results. Finally, like LOY, clonal hematopoiesis increases with increasing age and was very recently shown to associate with adverse outcome in CKD.^41^ Unfortunately, clonal hematopoiesis has not been assessed in the 2 cohorts of the present study, thus, we cannot comment on a potential interaction of LOY with clonal hematopoiesis.

In summary, LOY represents a novel risk factor for CVD in patients with CKD. It may explain high residual cardiovascular risk of CKD patients and, in particular, the higher incidence of heart failure in men. It should be the subject of future research to identify therapies to specifically target LOY-driven heart failure as a personalized sex-specific treatment approach in a steadily growing population of the elderly.

## ARTICLE INFORMATION

### Sources of Funding

Dr Speer is supported by the Else Kroener-Fresenius Foundation and the Deutsche Forschungsgemeinschaft (SFB TRR 219, project ID 322900939). Dr Dimmeler is supported by the Hessian Ministry for Science and the Arts (ENABLE) and the Leistungszentrum TheraNova. Dr. Speer and Dr Zeiher are supported by the Deutsche Forschungsgemeinschaft (FOR 5643/1, DI 600/12-1). Dr Zeiher is supported by the European Union (ERC-2021-ADG, GAP - 101054899, CHIP AVS). Views and opinions expressed are however those of the authors only and do not necessarily reflect those of the European Union or the European Research Council. Neither the European Union nor the granting authority can be held responsible for them. Dr Cremer is supported by the Deutsche Forschungsgemeinschaft (SFB1531, project code 456687919).

### Disclosures

Dr Speer receives speaker fees and honoraria from Amgen, Boehringer-Ingelheim, Astellas, Bayer, GSK, Novartis, NovoNordisk, Sanofi, Vifor, which are not related to the present study. Dr Wanner receives honoraria from Amgen, Astellas, AstraZeneca, Bayer, Boehringer Ingelheim, CSL-Vifor, FMC, Eli Lilly, GSK, Novartis, NovoNordisk, Sanofi. Dr Dimmeler is a scientific advisor for Pfizer. Dr Zeiher is a member of the scientific advisory board of Astra Zeneca, Boehringer Ingelheim, and TenSixteen Bio.

### Supplemental Material

Tables S1–S15

Figure S1

## Supplementary Material


