# Supplementary material for: Loss of Y Chromosome and Cardiovascular Events in Chronic Kidney Disease
Source: Circulation. 2024 Sep 3;150(10):746–57. doi: 10.1161/CIRCULATIONAHA.124.069139 (PMC11361358; doi:10.1161/CIRCULATIONAHA.124.069139)
Supplement: Supplementary file 1 [file cir-150-746-s001.pdf]

SUPPLEMENTAL MATERIAL

Supplemental Tables

Table S1
Association between LOY as continuous variable and mortality in participants of the CARE for HOMe study

Model	HR (95 % CI)	P
Crude	1.02 (1.01-1.03)	0.0002
Model 1	1.02 (1.01-1.03)	0.005
Model 2	1.02 (1.01-1.03)	0.008

Model 1: adjusted for age, systolic blood pressure, BMI, diabetes, smoking, hsCRP, NT-proBNP, troponin T, coronary artery disease
Model 2: adjusted for age, systolic blood pressure, BMI, diabetes, smoking, hsCRP, NT-proBNP, troponin T, coronary artery disease, eGFR, albuminuria, urinary DKK3

Table S2
Association between LOY and mortality in participants of the CARE for HOMe study

Model	LOY	No Event	Event	HR (95 % CI)	P
Crude	≤17 %	199 (80.2%)	49 (19.8%)	Reference	
	>17 %	13 (41.9%)	18 (58.1%)	3.21 (1.82-5.68)	<0.0001
Model 1	≤17 %	199 (80.2%)	49 (19.8%)	Reference	
	>17 %	13 (41.9%)	18 (58.1%)	2.31 (1.25-4.23)	0.007
Model 2	≤17 %	199 (80.2%)	49 (19.8%)	Reference	
	>17 %	13 (41.9%)	18 (58.1%)	2.58 (1.33-5.03)	0.005

Model 1: adjusted for age, systolic blood pressure, BMI, diabetes, smoking, hsCRP, NT-proBNP, troponin T, coronary artery disease
Model 2: adjusted for age, systolic blood pressure, BMI, diabetes, smoking, hsCRP, NT-proBNP, troponin T, coronary artery disease, eGFR, albuminuria, urinary DKK3

IDI for the addition of LOY to a model comprising age, systolic blood pressure, BMI, diabetes, smoking, hsCRP, NT-proBNP, troponin T, coronary artery disease, eGFR, albuminuria, and urinary DKK3: 0.304 (SE: 0.037), P<0.0001
NRI for the addition of LOY to a model comprising age, systolic blood pressure, BMI, diabetes, smoking, hsCRP, NT-proBNP, troponin T, coronary artery disease, eGFR, albuminuria, and urinary DKK3: 0.282 (SE: 0.084), P=0.0008

Table S3
Association between LOY and the combined cardiovascular endpoint in participants of the CARE for HOMe study

Model	LOY	No Event	Event	HR (95 % CI)	P
Crude	≤17 %	171 (69.0%)	77 (31.0%)	Reference	
	>17 %	10 (32.3%)	21 (67.7%)	2.14 (1.29-3.55)	0.003
Model 1	≤17 %	171 (69.0%)	77 (31.0%)	Reference	
	>17 %	10 (32.3%)	21 (67.7%)	1.72 (1.00-2.96)	0.049
Model 2	≤17 %	171 (69.0%)	77 (31.0%)	Reference	
	>17 %	10 (32.3%)	21 (67.7%)	1.82 (1.04-3.20)	0.037

Model 1: adjusted for age, systolic blood pressure, BMI, diabetes, smoking, hsCRP, NT-proBNP, troponin T, coronary artery disease
Model 2: adjusted for age, systolic blood pressure, BMI, diabetes, smoking, hsCRP, NT-proBNP, troponin T, coronary artery disease, eGFR, albuminuria, urinary DKK3

IDI for the addition of LOY to a model comprising age, systolic blood pressure, BMI, diabetes, smoking, hsCRP, NT-proBNP, troponin T, coronary artery disease, eGFR, albuminuria, and urinary DKK3: 0.120 (SE: 0.020), P<0.0001
NRI for the addition of LOY to a model comprising age, systolic blood pressure, BMI, diabetes, smoking, hsCRP, NT-proBNP, troponin T, coronary artery disease, eGFR, albuminuria, and urinary DKK3: 0.309 (SE: 0.073), P<0.0001

Table S4
Association between LOY and decompensation of heart failure or death in participants of the CARE for HOMe study

Model	LOY	No Event	Event	HR (95 % CI)	P
Crude	≤17 %	182 (73.4%)	66 (26.6%)	Reference	
	>17 %	12 (38.7%)	19 (61.3%)	2.77 (1.63-4.72)	0.0002
Model 1	≤17 %	182 (73.4%)	66 (26.6%)	Reference	
	>17 %	12 (38.7%)	19 (61.3%)	2.04 (1.14-3.66)	0.016
Model 2	≤17 %	182 (73.4%)	66 (26.6%)	Reference	
	>17 %	12 (38.7%)	19 (61.3%)	2.30 (1.23-4.27)	0.009

Model 1: adjusted for age, systolic blood pressure, BMI, diabetes, smoking, hsCRP, NT-proBNP, troponin T, coronary artery disease
Model 2: adjusted for age, systolic blood pressure, BMI, diabetes, smoking, hsCRP, NT-proBNP, troponin T, coronary artery disease, eGFR, albuminuria, urinary DKK3

IDI for the addition of LOY to a model comprising age, systolic blood pressure, BMI, diabetes, smoking, hsCRP, NT-proBNP, troponin T, coronary artery disease, eGFR, albuminuria, and urinary DKK3: 0.134 (SE: 0.021), P<0.0001
NRI for the addition of LOY to a model comprising age, systolic blood pressure, BMI, diabetes, smoking, hsCRP, NT-proBNP, troponin T, coronary artery disease, eGFR, albuminuria, and urinary DKK3: 0.407 (SE: 0.086), P<0.0001

Table S5
Association between LOY and decompensation of heart failure in participants of the CARE for HOMe study

Model	LOY	No Event	Event	HR (95 % CI)	P
Crude	≤17 %	218 (87.9%)	30 (12.1%)	Reference	
	>17 %	21 (67.7%)	10 (32.3%)	3.11 (1.49-6.49)	0.0026
Model 1	≤17 %	218 (87.9%)	30 (12.1%)	Reference	
	>17 %	21 (67.7%)	10 (32.3%)	2.52 (1.13-5.63)	0.024
Model 2	≤17 %	218 (87.9%)	30 (12.1%)	Reference	
	>17 %	21 (67.7%)	10 (32.3%)	2.73 (1.15-6.46)	0.022

Model 1: adjusted for age, systolic blood pressure, BMI, diabetes, smoking, hsCRP, NT-proBNP, troponin T, coronary artery disease
Model 2: adjusted for age, systolic blood pressure, BMI, diabetes, smoking, hsCRP, NT-proBNP, troponin T, coronary artery disease, eGFR, albuminuria, urinary DKK3

Table S6
Association between LOY and decompensation of heart failure or death in participants of the CARE for HOMe study

**Model**	**LOY**	**HR (95 % CI)**	**P**
Crude	≤17 %	Reference	
	>17 %	2.85 (1.08-7.50)	0.034
Model 1	≤17 %	Reference	
	>17 %	3.80 (1.08-13.29)	0.037

Model 1: adjusted for age, systolic blood pressure, BMI, diabetes, smoking, hsCRP, NT-proBNP, troponin T, coronary artery disease, eGFR, albuminuria, urinary DKK3, EF, and LVM

Table S7
Association between LOY and the combined kidney endpoint in participants of the CARE for HOMe study

Model	LOY	No Event	Event	HR (95 % CI)	P
Crude	≤17 %	206 (83.1%)	42 (16.9%)	Reference	
	>17 %	27 (87.1%)	4 (12.9%)	0.83 (0.30-2.33)	0.73
Model 1	≤17 %	206 (83.1%)	42 (16.9%)	Reference	
	>17 %	27 (87.1%)	4 (12.9%)	0.52 (0.17-1.59)	0.25
Model 2	≤17 %	206 (83.1%)	42 (16.9%)	Reference	
	>17 %	27 (87.1%)	4 (12.9%)	0.67 (0.22-2.03)	0.48

Model 1: adjusted for age, systolic blood pressure, BMI, diabetes, smoking, hsCRP, NT-proBNP, troponin T, coronary artery disease
Model 2: adjusted for age, systolic blood pressure, BMI, diabetes, smoking, hsCRP, NT-proBNP, troponin T, coronary artery disease, eGFR, albuminuria, urinary DKK3

Table S8
Association between LOY and the end-stage kidney disease in participants of the CARE for HOMe study

Model	LOY	No Event	Event	HR (95 % CI)	P
Crude	≤17 %	210 (84.7%)	38 (15.3%)	Reference	
	>17 %	27 (87.1%)	4 (12.9%)	0.95 (0.34-2.68)	0.93
Model 1	≤17 %	210 (84.7%)	38 (15.3%)	Reference	
	>17 %	27 (87.1%)	4 (12.9%)	0.59 (0.19-1.85)	0.37
Model 2	≤17 %	210 (84.7%)	38 (15.3%)	Reference	
	>17 %	27 (87.1%)	4 (12.9%)	0.89 (0.29-2.76)	0.85

Model 1: adjusted for age, systolic blood pressure, BMI, diabetes, smoking, hsCRP, NT-proBNP, troponin T, coronary artery disease
Model 2: adjusted for age, systolic blood pressure, BMI, diabetes, smoking, hsCRP, NT-proBNP, troponin T, coronary artery disease, eGFR, albuminuria, urinary DKK3

Table S9
Baseline characteristics according to categories of LOY in participants of the 4D study

	LOY ≤17 % N=506	LOY >17 % N=38	P
**Age (years)**	64.4±8.3	68.8±6.6	0.002
**BMI (kg/m^2)**	27.1±4.3	26.1±3.3	0.16
**CAD (%)**	26.3	23.7	0.85
**Congestive heart failure (%)**	31.2	52.6	0.011
**Smoking (%)**	12.5	18.4	0.026
**Systolic blood pressure (mmHg)**	145.5±21.0	145.7±23.2	0.96
**LDL-C (mg/dL)**	120.0±27.4	121.7±28.2	0.76
**NT-proBNP (pg/mL)**	3047.5 (8244.5)	5065.0 (4747.0)	0.20
**Troponin T (ng/mL)**	67.0 (7.0)	65.0 (6.0)	0.69
**hsCRP (mg/L)**	5.6 (8.5)	9.4 (7.3)	0.29
**Hemoglobin (g/dL)**	11.1±1.4	11.1±1.5	0.99
**Glycosylated hemoglobin (%)**	6.6±1.2	6.6±1.4	0.77
**Albumin (g/L)**	3.9±0.3	3.9±0.3	0.38
**Time receiving dialysis (months)**	5.5 (8.3)	7.8 (7.2)	0.13
**LOY (%)**	0.2 (8.9)	29.3 (38.1)	<0.001
**Therapy (% atorvastatin)**	48.8	50.0	1.0

Table S10
Association between LOY and mortality in participants of the 4D study

**Model**	**LOY**	**No Event**	**Event**	**HR (95 % CI)**	**P**
Crude	≤17 %	354 (70.0%)	152 (30.0%)	Reference	
	>17 %	7 (18.4%)	31 (81.6%)	3.34 (2.14-5.21)	<0.0001
Model 1	≤17 %	354 (70.0%)	152 (30.0%)	Reference	
	>17 %	7 (18.4%)	31 (81.6%)	2.75 (1.83-4.17)	<0.0001
Model 2	≤17 %	354 (70.0%)	152 (30.0%)	Reference	
	>17 %	7 (18.4%)	31 (81.6%)	2.76 (1.83-4.16)	<0.0001

Model 1: adjusted for age, systolic blood pressure, BMI, smoking, hsCRP, troponin T, NT-proBNP, coronary artery disease, congestive heart failure
Model 2: adjusted for age, systolic blood pressure, BMI, smoking, hsCRP, troponin T, NT-proBNP, coronary artery disease, congestive heart failure, randomization

IDI for the addition of LOY to a model comprising age, systolic blood pressure, BMI, smoking, hsCRP, NT-proBNP, troponin T, coronary artery disease, congestive heart failure, randomization group: 0.094 (SE: 0.013), P<0.0001
NRI for the addition of LOY to a model comprising age, systolic blood pressure, BMI, smoking, hsCRP, NT-proBNP, troponin T, coronary artery disease, congestive heart failure, randomization group: 0.131 (SE: 0.032), P=0.0006

Table S11
Association between LOY as continuous variable and mortality in participants of the 4D study

Model	HR (95 % CI)	P
Crude	1.02 (1.01-1.03)	0.001
Model 1	1.01 (1.00-1.02)	0.031
Model 2	1.01 (1.00-1.02)	0.030

Model 1: adjusted for age, systolic blood pressure, BMI, smoking, hsCRP, NT-proBNP, troponin T, coronary artery disease, congestive heart failure
Model 2: adjusted for age, systolic blood pressure, BMI, smoking, hsCRP, NT-proBNP, troponin T, coronary artery disease, congestive heart failure, randomization

Table S12
Association between LOY and the combined primary cardiovascular endpoint in participants of the 4D study

**Model**	**LOY**	**No Event**	**Event**	**HR (95 % CI)**	**P**
Crude	≤17 %	363 (71.7%)	143 (28.3%)	Reference	
	>17 %	13 (34.2%)	25 (65.8%)	2.48 (1.50-4.12)	<0.0001
Model 1	≤17 %	363 (71.7%)	143 (28.3%)	Reference	
	>17 %	13 (34.2%)	25 (65.8%)	2.77 (1.76-4.35)	<0.0001
Model 2	≤17 %	363 (71.7%)	143 (28.3%)	Reference	
	>17 %	13 (34.2%)	25 (65.8%)	2.77 (1.76-4.36)	<0.0001

Model 1: adjusted for age, systolic blood pressure, BMI, smoking, hsCRP, NT-proBNP, troponin T, coronary artery disease, congestive heart failure
Model 2: adjusted for age, systolic blood pressure, BMI, smoking, hsCRP, NT-proBNP, troponin T, coronary artery disease, congestive heart failure, randomization

IDI for the addition of LOY to a model comprising age, systolic blood pressure, BMI, smoking, hsCRP, NT-proBNP, troponin T, coronary artery disease, congestive heart failure, randomization group: 0.061 (SE: 0.013), P<0.0001
NRI for the addition of LOY to a model comprising age, systolic blood pressure, BMI, smoking, hsCRP, NT-proBNP, troponin T, coronary artery disease, congestive heart failure, randomization group: 0.142 (SE: 0.038), P=0.0005

Table S13
Association between LOY and the combined endpoint of sudden cardiac death and death due to heart failure in participants of the 4D study

Model	LOY	No Event	Event	HR (95 % CI)	P	P*
Crude	≤17 %	459 (90.7%)	47 (9.3%)	Reference		
	>17 %	26 (68.4%)	12 (31.6%)	4.15 (2.08-8.26)	<0.0001	<0.0001
Model 1	≤17 %	459 (90.7%)	47 (9.3%)	Reference		
	>17 %	26 (68.4%)	12 (31.6%)	4.10 (2.09-8.05)	<0.0001	<0.0001
Model 2	≤17 %	459 (90.7%)	47 (9.3%)	Reference		
	>17 %	26 (68.4%)	12 (31.6%)	4.11 (2.09-8.08)	<0.0001	<0.0001

Model 1: adjusted for age, systolic blood pressure, BMI, smoking, hsCRP, NT-proBNP, troponin T, coronary artery disease, congestive heart failure
Model 2: adjusted for age, systolic blood pressure, BMI, smoking, hsCRP, NT-proBNP, troponin T, coronary artery disease, congestive heart failure, randomization

IDI for the addition of LOY to a model comprising age, systolic blood pressure, BMI, smoking, hsCRP, NT-proBNP, troponin T, coronary artery disease, congestive heart failure, randomization group: 0.021 (SE: 0.006), P=0.0017
NRI for the addition of LOY to a model comprising age, systolic blood pressure, BMI, smoking, hsCRP, NT-proBNP, troponin T, coronary artery disease, congestive heart failure, randomization group: 0.141 (SE: 0.037), P=0.0004

P* = Bonferroni corrected p-value

Table S14
Association between LOY and the combined coronary endpoint in participants of the 4D study

**Model**	**LOY**	**No Event**	**Event**	**HR (95 % CI)**	**P**	**P***
Crude	≤17 %	382 (75.5%)	124 (24.5%)	Reference		
	>17 %	26 (68.4%)	12 (31.6%)	1.38 (0.67-2.82)	0.38	0.77
Model 1	≤17 %	382 (75.5%)	124 (24.5%)	Reference		
	>17 %	26 (68.4%)	12 (31.6%)	1.69 (0.92-3.13)	0.09	0.18
Model 2	≤17 %	382 (75.5%)	124 (24.5%)	Reference		
	>17 %	26 (68.4%)	12 (31.6%)	1.67 (0.92-3.13)	0.09	0.18

Model 1: adjusted for age, systolic blood pressure, BMI, smoking, hsCRP, NT-proBNP, troponin T, coronary artery disease, congestive heart failure
Model 2: adjusted for age, systolic blood pressure, BMI, smoking, hsCRP, NT-proBNP, troponin T, coronary artery disease, congestive heart failure, randomization

P* = Bonferroni corrected p-value

Table S15
Association between LOY and all-cause mortality in participants of the 4D study according to randomization group

**Model**		**Placebo group**		**Atorvastatin group**	
	**LOY**	**HR (95% CI)**	**P**	**HR (95% CI)**	**P**
Crude	≤17 %	Reference		Reference	
	>17 %	3.91 (2.20-6.98)	<0.0001	2.67 (1.32-5.38)	0.006
Model 1	≤17 %	Reference		Reference	
	>17 %	2.79 (1.57-4.95)	0.0005	2.73 (1.50-4.98)	0.001

Model 1: adjusted for age, systolic blood pressure, BMI, smoking, hsCRP, NT-proBNP, troponin T, coronary artery disease, congestive heart failure
Interaction term between randomization group and LOY: P=0.89

Supplemental figures

Figure S1

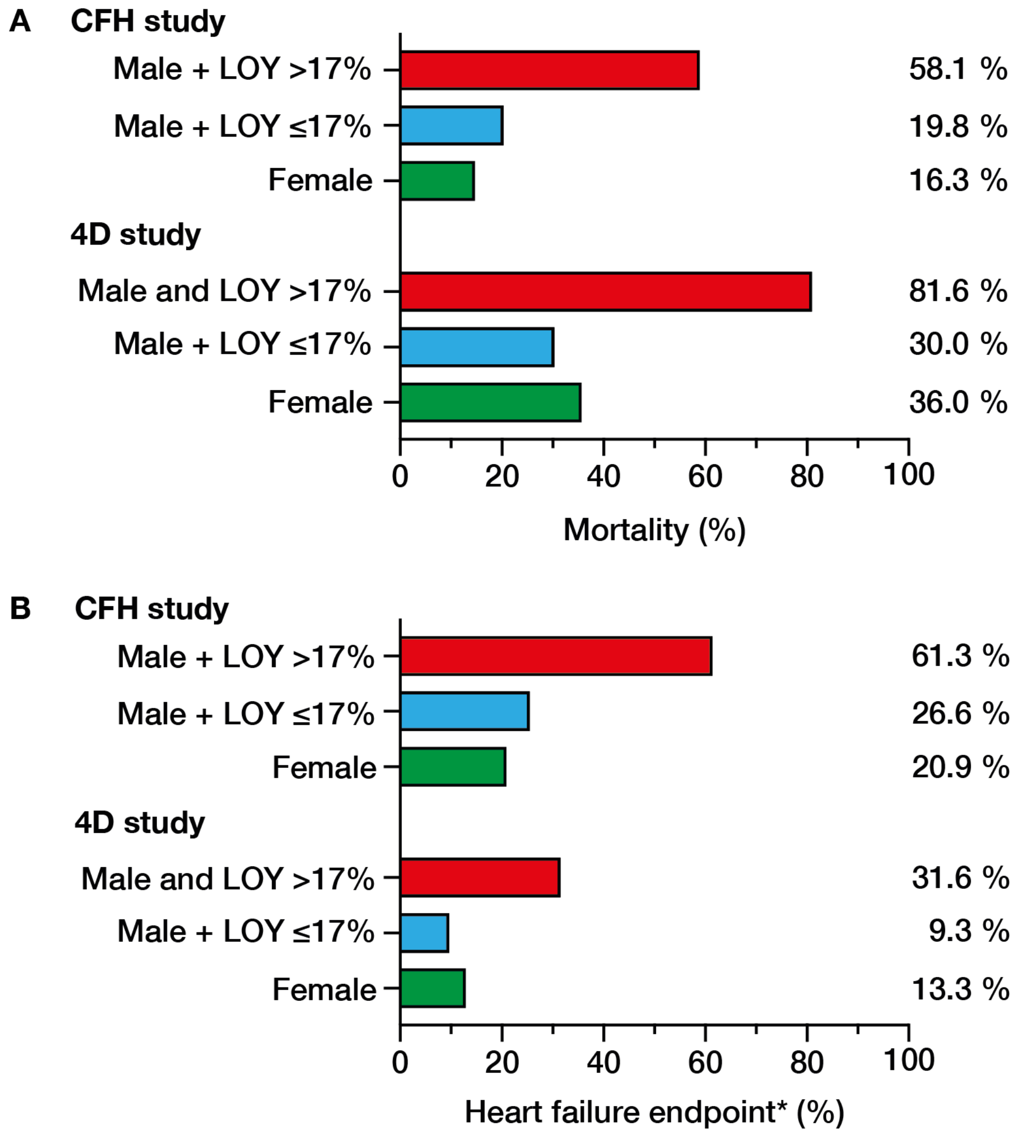

Incidence of **(A)** mortality and the **(B)** heart failure endpoint in females, males with LOY ≤17 %, males with LOY >17 % of the CARE for HOMe and 4D study.

* Heart failure endpoint in CARE for HOMe: composite of cardiac decompensation and death; in 4D: composite of death due to heart failure and sudden cardiac death.
